# Rgs2 Mediates Pro-Angiogenic Function of Myeloid Derived Suppressor Cells in the Tumor Microenvironment via Upregulation of MCP-1

**DOI:** 10.1371/journal.pone.0018534

**Published:** 2011-04-11

**Authors:** Kimberly C. Boelte, Laura E. Gordy, Sebastian Joyce, Mary Ann Thompson, Li Yang, P. Charles Lin

**Affiliations:** 1 Department of Cancer Biology, Vanderbilt University Medical Center, Nashville, Tennessee, United States of America; 2 Department of Microbiology and Immunology, Vanderbilt University Medical Center, Nashville, Tennessee, United States of America; 3 Department of Pathology, Vanderbilt University Medical Center, Nashville, Tennessee, United States of America; 4 Center for Cancer Research, National Institutes of Health, Bethesda, Maryland, United States of America; University of Southern California, United States of America

## Abstract

**Background:**

Tumor growth is intimately linked with stromal interactions. Myeloid derived suppressor cells (MDSCs) are dramatically elevated in cancer patients and tumor bearing mice. MDSCs modulate the tumor microenvironment through attenuating host immune response and increasing vascularization.

**Methodology/Principal Findings:**

In searching for molecular mediators responsible for pro-tumor functions, we found that regulator of G protein signaling-2 (Rgs2) is highly increased in tumor-derived MDSCs compared to control MDSCs. We further demonstrate that hypoxia, a common feature associated with solid tumors, upregulates the gene expression. Genetic deletion of Rgs2 in mice resulted in a significant retardation of tumor growth, and the tumors exhibit decreased vascular density and increased cell death. Interestingly, deletion of Rgs2 in MDSCs completely abolished their tumor promoting function, suggesting that Rgs2 signaling in MDSCs is responsible for the tumor promoting function. Cytokine array profiling identified that Rgs2−/− tumor MDSCs produce less MCP-1, leading to decreased angiogenesis, which could be restored with addition of recombinant MCP-1.

**Conclusion:**

Our data reveal Rgs2 as a critical regulator of the pro-angiogenic function of MDSCs in the tumor microenvironment, through regulating MCP-1 production.

## Introduction

It has become clear that the tumor microenvironment plays an important role in tumor progression. Tumors are comprised of several host derived cell types, including fibroblasts, smooth muscle cells, endothelial cells, immune cells, and epithelial cells, each contributing to the microenvironment in ways we are only beginning to understand [1]. In addition to the cells present, the tumor microenvironment contains extracellular matrix (ECM) and other factors secreted by the tumor and stromal cells that can greatly affect tumor progression.

Immune suppression and promotion of angiogenesis are essential for tumor growth and progression. Interestingly, MDSCs possess both properties, and create an environment to facilitate tumor progression. MDSCs increase in tumor bearing hosts, including cancer patients, and this accumulation is mediated by inflammatory and angiogenic factors [2]. MDSCs are also known to promote a shift to a type 2, tumor-promoting response in macrophages [3]. In addition, they infiltrate into tumors, and promote tumor vascularization, tumor growth, and metastasis through modulating VEGF bioavailability and protease activity in the tumor microenvironment [4], [5], [6], [7]. The pro-angiogenic function of these myeloid cells is sufficient to confer tumor refractoriness to anti-VEGF treatment [8], a common target for anti-angiogenic therapy. This further illustrates the importance of MDSCs in tumor progression, as well as in molecular therapies for cancer.

Rgs2 (NM_009061) is a signaling molecule known to function downstream of G protein coupled receptors. Rgs2 contains a conserved Regulator of G protein Signaling domain, and functions as a GTPase-activating protein (GAP) for several Gα subunits of G proteins [9], [10], [11]. Rgs2 is widely expressed in a variety of cells, including myeloid cells [12], [13]. A variety of stimuli can induce Rgs2 expression, most of which signal through G proteins. Therefore, Rgs2 functions in a negative feedback loop with regard to G protein coupled receptors (GPCRs). It enhances the intrinsic GTPase activity of the Gα subunit, and thereby decreases the time that the G protein subunits are dissociated, leading to decreased signaling [14], [15]. In addition, cell stress, such as heat shock or DNA damage, can also increase Rgs2 levels [16], [17]. Rgs2 inhibits cell proliferation, and is a known mediator of cell differentiation in several cell types, such as myeloid cells [18].

Monocyte chemoattractant protein 1 (MCP-1) is a chemokine important for cell migration [19], [20]. It signals through CCR2, a GPCR found on monocytes, endothelial cells, T cells, etc. [20], [21], [22]. In part due to a migratory effect on endothelial cells, MCP-1 is a potent angiogenic factor, promoting vascularization *ex vivo* and *in vivo* [22]. Blocking MCP-1 with a neutralizing antibody inhibited angiogenesis, and led to decreased tumor metastases and increased survival in a mouse tumor model [22].

Here, we report a novel role of Rgs2 in tumor growth and progression. Rgs2 expression is elevated in tumor derived MDSCs, and hypoxia, a condition commonly associated with tumors, upregulates its expression. Inactivation of Rgs2 in MDSCs leads to a significant reduction of MCP-1, and retards tumor angiogenesis and tumor progression. Thus, this study identifies Rgs2 as a critical mediator of pro-angiogenic function associated with MDSCs in the tumor microenvironment.

## Materials and Methods

### Ethics

All mouse studies have been conducted according to Animal Welfare Act and the Public Health Service Policy and approved by Vanderbilt University Institution Animal Care and Use Committee (IACUC) (M/05/083). The animals were housed in pathogen-free units at Vanderbilt University Medical Center, in compliance with IACUC regulations. Rgs2−/− mice in C57Bl/6 background were obtained from Dr. Josef Penninger at the Institute of Molecular Biotechnology GmbH, and were bred at Vanderbilt University Medical Center. Age and gender matched wild type control mice were purchased from Jackson Laboratories.

### Cell culture

HL-60 and 3LL cell lines (purchased from ATCC) were cultured in RPMI 1640+10% FBS at 37°C, 5% CO_2_. The 3LL cell line is a subclone of LLC (Lewis lung carcinoma) cell line. The B16 (purchased from ATCC) and MC26 [4] cell lines were grown in DMEM+10% FBS. Human umbilical vein endothelial cells (HUVECs) were obtained from Lonza, and cultured in EGM-2 Bulletkit medium, also from Lonza, at 37°C, 5% CO_2_. HUVECs were used between passages 3 to 7.

### Isolation of lung microvascular cells

Mice were sacrificed, and the chest cavity was opened. The right ventricle of the heart was punctured with a 25 gauge butterfly needle and syringe. The heart and lungs were flushed with PBS+2 mM EDTA until the lungs were white, followed by 0.25% trypsin + 2 mM EDTA until the lungs were pink. Lungs were removed, pumped with more trypsin, and allowed to incubate at 37°C for 20 minutes. The lungs were then diced, and the tissue was pipetted several times in DMEM + 10% FBS. After centrifugation, the cell pellet was resuspended in EGM, and seeded into plates. Medium was changed the next day to remove cell debris and red blood cells.

### RT-PCR

RNA was isolated from cells using the RNeasy kit from Qiagen. iScript cDNA synthesis kit (BioRad) was used to produce cDNA. For PCR, Hi-Fidelity PCR mix (Invitrogen) was used, and for Real Time PCR, SYBR green kit (BioRad) and an iCycler or MyiQ machine (BioRad) were used.

### Tumor model

For tumor growth studies, 3LL or B16 cells were injected subcutaneously into the left hindlimbs of mice. For the reconstitution assay, 3LL cells were mixed with MDSCs isolated by fluorescence activated cell sorting (FACS) and injected as before. Tumor size was measured by caliper, and tumor volume was calculated as volume = length×(width)^2^×0.5. Tumor samples taken at days 17–21 post-injection were flash frozen in OCT (Sakura) or fixed in formalin and embedded in paraffin, then sectioned. Sections were incubated with primary antibodies overnight, then either Texas Red-fluorescent or biotin-labeled secondary antibodies for immunofluorescent or immunohistochemical analysis, respectively. Slides were imaged on an Olympus BX51 microscope with an Olympus DP70 camera. Images were overlaid using Adobe Photoshop.

### Flow cytometry, FACS, and MACS

Tissues were prepared into single cell suspensions and labeled with antibodies for markers of mature and immature blood cells (BD Biosciences). Cells were analyzed using a BD LSRII or BD FACScan. For FACS, spleen cells were labeled with Gr-1 (Miltenyi Biotec) and CD11b (BD Biosciences) and sorted in the VA Flow Cytometry Resource.

**Figure 1:**
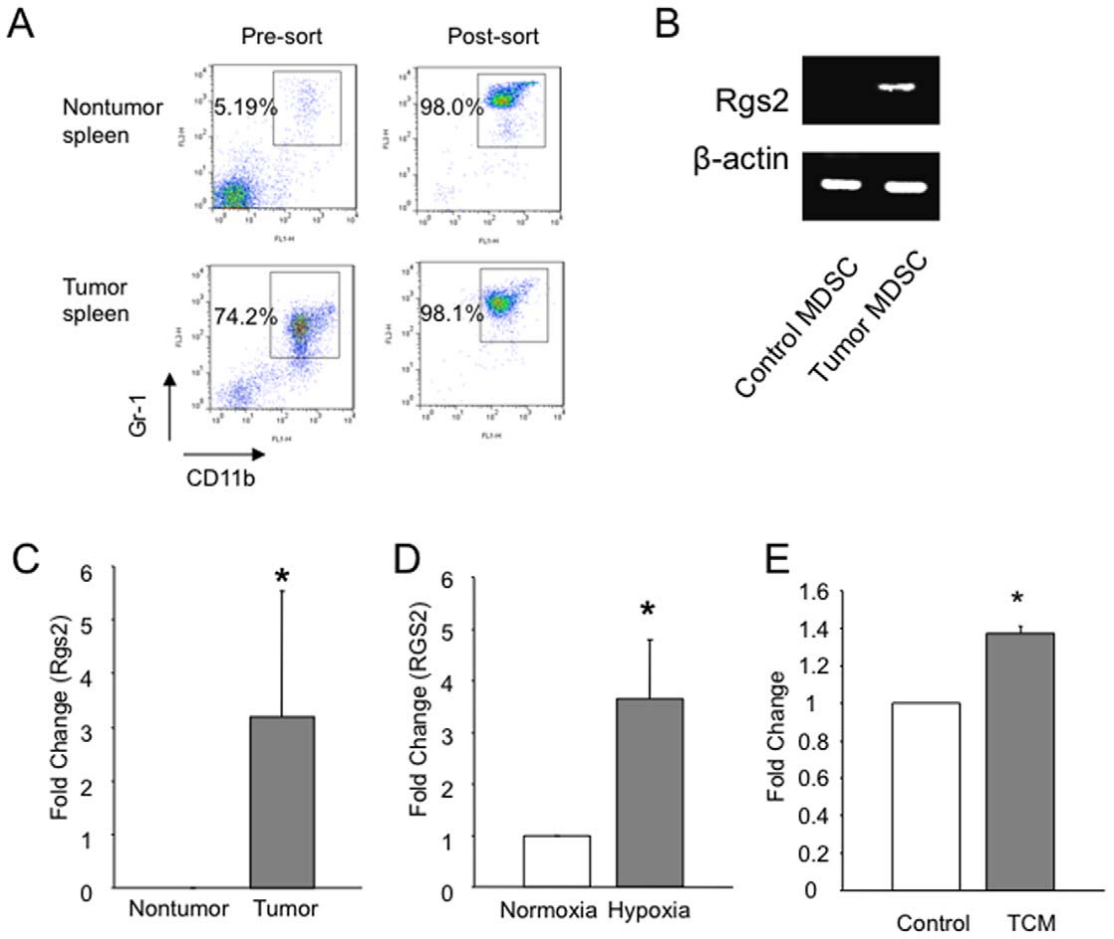
Induction of Rgs2 in tumor derived MDSCs. (A) Purity of cells from isolation of Gr-1+CD11b+ cells using MACS. Spleens from non-tumor bearing and MC26 tumor bearing BALB/c mice were isolated and processed into to single cell suspension, followed by sorting using MACS, as described in the Methods section. (B) and (C) Gr-1+CD11b+ cells were isolated from non tumor bearing and tumor bearing mice, MC26 tumors in BALB/c (B) and 3LL tumors in C57BL/6 (C), by magnetic sorting of pooled splenocytes from 5–10 mice, generating MDSCs of greater than 98% purity. Cells were then subjected to RNA isolation and RT-PCR (B) and real time PCR (C) for Rgs2 expression. These experiments were repeated 3 times. (D) HL-60 cells were incubated under normoxic (20% O_2_) or hypoxic (1.0–2% O_2_) conditions for an hour, and RNA was isolated, followed by real time PCR analysis. This experiment was performed 5 times. (E) HL-60 cells were treated with medium conditioned by 3LL tumor cells for one hour. RNA was isolated, followed by real time PCR analysis. The experiment was performed 3 times. * p<0.05.

For magnetic activated cell sorting (MACS) of MDSCs, tumors were digested with collagenase A (Sigma) and hyaluronidase (Sigma). Tumor and spleen MDSCs were isolated by sequential labeling and column isolation with anti-Gr1-PE and anti-PE multisort beads, then CD11b beads (all from Miltenyi). We achieved equal to or greater than 98% purity of Gr-1+CD11b+ cells using MACS (Figure 1A).

### BrdU incorporation

BrdU incorporation was performed using the BrdU Flow Kit from BD Biosciences per the manufacturer's instructions. Briefly, BrdU was added to endothelial cells at a final concentration of 10 uM. The cells were collected after 2 hours, fixed, and permeabilized. The cells were then subjected to DNase treatment at 37°C, followed by labeling with anti-BrdU and analysis by flow cytometry.

### MDSC morphology

MDSCs were isolated from spleens of wild type and Rgs2−/− tumor bearing mice by MACS. The samples were placed on a slide using a cytospin centrifuge, stained with the Three Step Stain (Richard Allan Scientific), and analyzed for morphology by a hematopathologist (M.A. Thompson) in blind fashion.

### In vitro angiogenic assays

Endothelial cell migration: Tumor MDSCs were isolated by MACS and cultured in RPMI with 1% FBS overnight. Transwells with 8 micron pores (Corning) were coated with fibronectin in 0.1% gelatin at 37°C one hour prior to addition of HUVECs. Transwells were placed over the cultured MDSCs, and HUVECs were added in basal RPMI at 1×10^5^/well and allowed to migrate for 3.5 hours. Migrated HUVECs were fixed, stained with crystal violet, and counted in randomly selected fields under microscopy.

Vascular tube formation: MDSCs were isolated by MACS from tumor tissue, and cultured in EBM (Lonza) plus 1% FBS overnight. Wells of 48-well plates were coated with Matrigel (BD Bioscience). HUVECs were suspended in MDSC conditioned medium and plated over Matrigel at 80,000 cells/well. Tube formation was scored by counting branch points at 48 and 72 hours.

### Cytokine array

MDSCs were isolated from tumor tissues using MACS, and the cytokine array was performed according to the manufacturer's protocol (RayBiotech). The data were analyzed by densitometry, with each band being normalized to internal controls.

### Statistical analysis

Data were averaged and compared using Student's t test. Error bars on graphs represent standard error across experiments or mice.

## Results

### Rgs2 is dramatically increased in tumor derived MDSCs

It is well documented that MDSCs from tumor bearing mice function differently from those from non-tumor bearing mice. In an effort to investigate the mechanism the tumor utilizes to condition the MDSCs, we examined gene expression on isolated MDSCs from spleens of BALB/c mice, either tumor bearing or non-tumor bearing. We achieved greater than 98% purity (Figure 1A). RT-PCR analysis showed a significant increase of Rgs2 mRNA in tumor derived MDSCs compared to cells from non-tumor hosts in both MC26 tumor model in BALB/c mice (Figure 1B) and 3LL tumor model in C57BL/6 mice (Figure 1C). Interestingly, we found a similar Rgs2 induction in MDSCs associated with MC26 and 3LL tumor conditions compared to non-tumor conditions by RT-PCR (Figure 1B–C). Further analysis showed that hypoxia, a feature commonly associated with solid tumors, significantly increased Rgs2 RNA levels in a myeloid cell line *in vitro* (Figure 1D), consistent with published studies indicating that Rgs2 may be a stress response gene, increasing rapidly under conditions which are stressful for the cell. In addition, treatment with 3LL tumor conditioned medium led to a significant increase in Rgs2 mRNA levels in myeloid cells, suggesting that the tumor cells are secreting a factor capable of modulating Rgs2 expression (Figure 1E). The findings demonstrate that tumor conditions, such as hypoxia and secreted factors, upregulate Rgs2 expression in MDSCs.

### Rgs2 signaling in MDSCs mediates tumor growth

**Figure 2:**
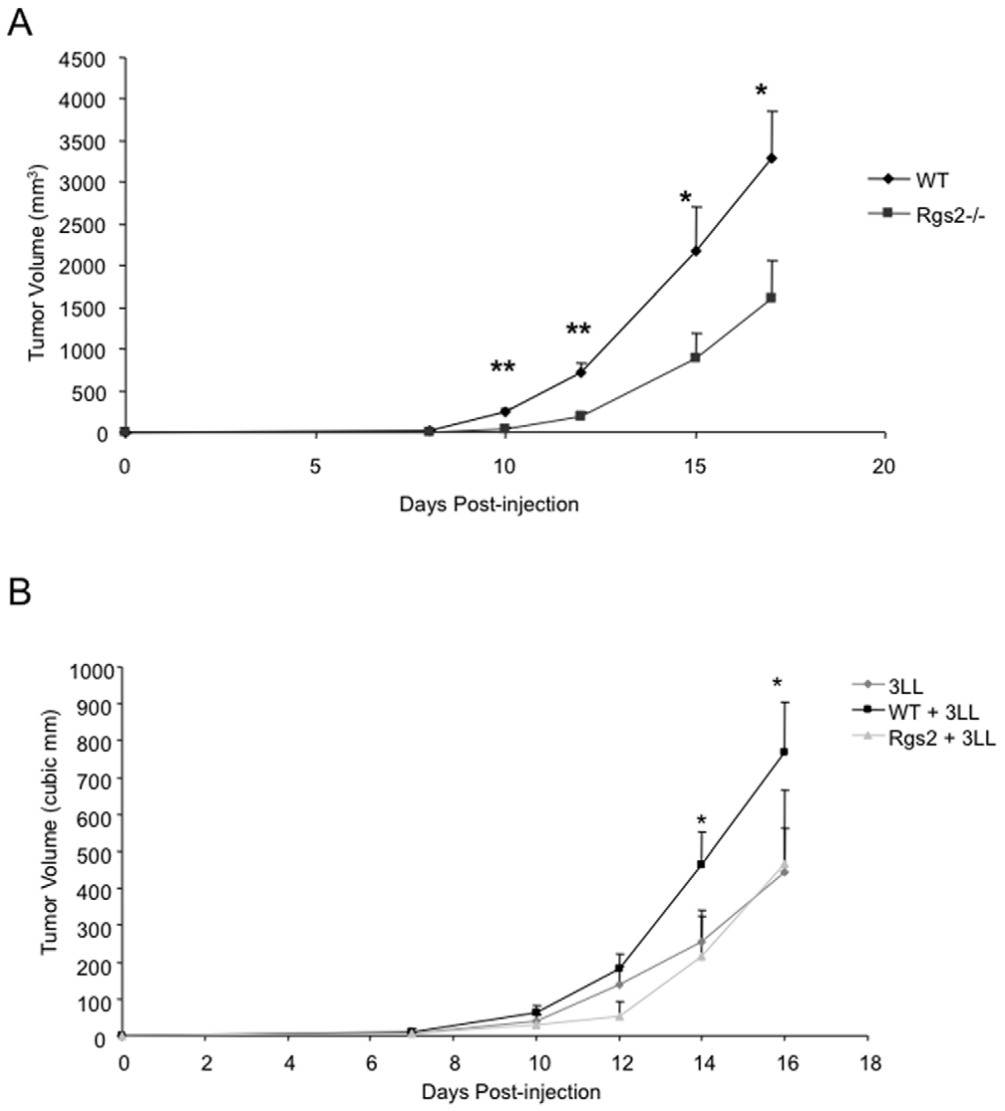
Deletion of Rgs2 in MDSCs retards tumor growth. (A) Rgs2−/− and C57BL/6 wild type mice were injected with 5×10^5^ 3LL tumor cells subcutaneously in the hindlimb, and tumor size was measured by caliper over time (n = 12 mice per group). This experiment was repeated 3 times. (B) Wild type mice were injected subcutaneously in the hindlimb with 1×10^5^ 3LL cells alone, or 3LL cells combined with 1×10^4^ wild type or Rgs2−/− MDSCs sorted by flow cytometry (>95% purity; data not shown) from spleens of tumor-bearing mice. Tumor growth was measured by caliper over time. n = 8 mice per group. This experiment was performed twice. * p<0.05.

Because MDSCs of tumor bearing mice upregulate Rgs2 expression, we sought to understand the role Rgs2 plays in MDSCs in tumor progression using Rgs2 knockout mice. Mice without Rgs2 are viable, healthy, and fertile, but have defects in hippocampal development [23]. Since the Rgs2 null mouse was developed in the C57Bl/6 background, we injected Rgs2−/− mice and C57BL/6 wild type controls with syngeneic 3LL cells subcutaneously in the hindlimb, and measured tumor growth over time. The null mice exhibited significantly retarded tumor growth compared to the wild type mice (Figure 2A). Similar results were achieved when the mice were injected with a syngeneic melanoma tumor line, B16 (data not shown). These results reveal a positive role of Rgs2 in tumor growth and progression.

**Figure 3:**
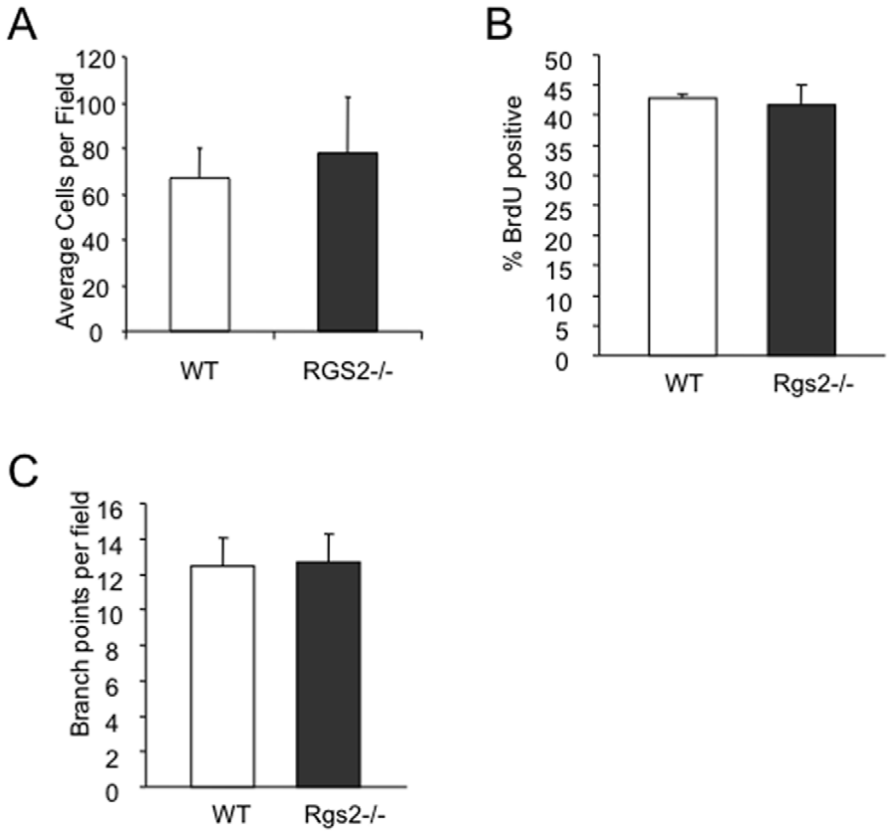
Lack of Rgs2 in endothelial cells does not affect migration, BrdU incorporation, or tube fomation. Lung microvascular endothelial cells were isolated from lungs of wild type and Rgs2−/− mice and cultured. (A) 1×10^5^ cells were placed in the top chamber of a Transwell, and migrated to growth medium for 4 hours. Cells on the bottom of the Transwell (migrated cells) were fixed, stained with crystal violet, and counted. Experiment was performed 3 times in duplicate. (B) Cells were pulsed with 10 uM BrdU for 2 hours, then collected, processed, and analyzed by flow cytometry. The average percentages of BrdU positive cells was plotted. Experiment was performed 2 times in duplicate. (C) Cells were seeded on top of Matrigel in growth medium and allowed to form tube structures for 48 hours. Branch points were counted, and the average number per field was graphed.

**Figure 4:**
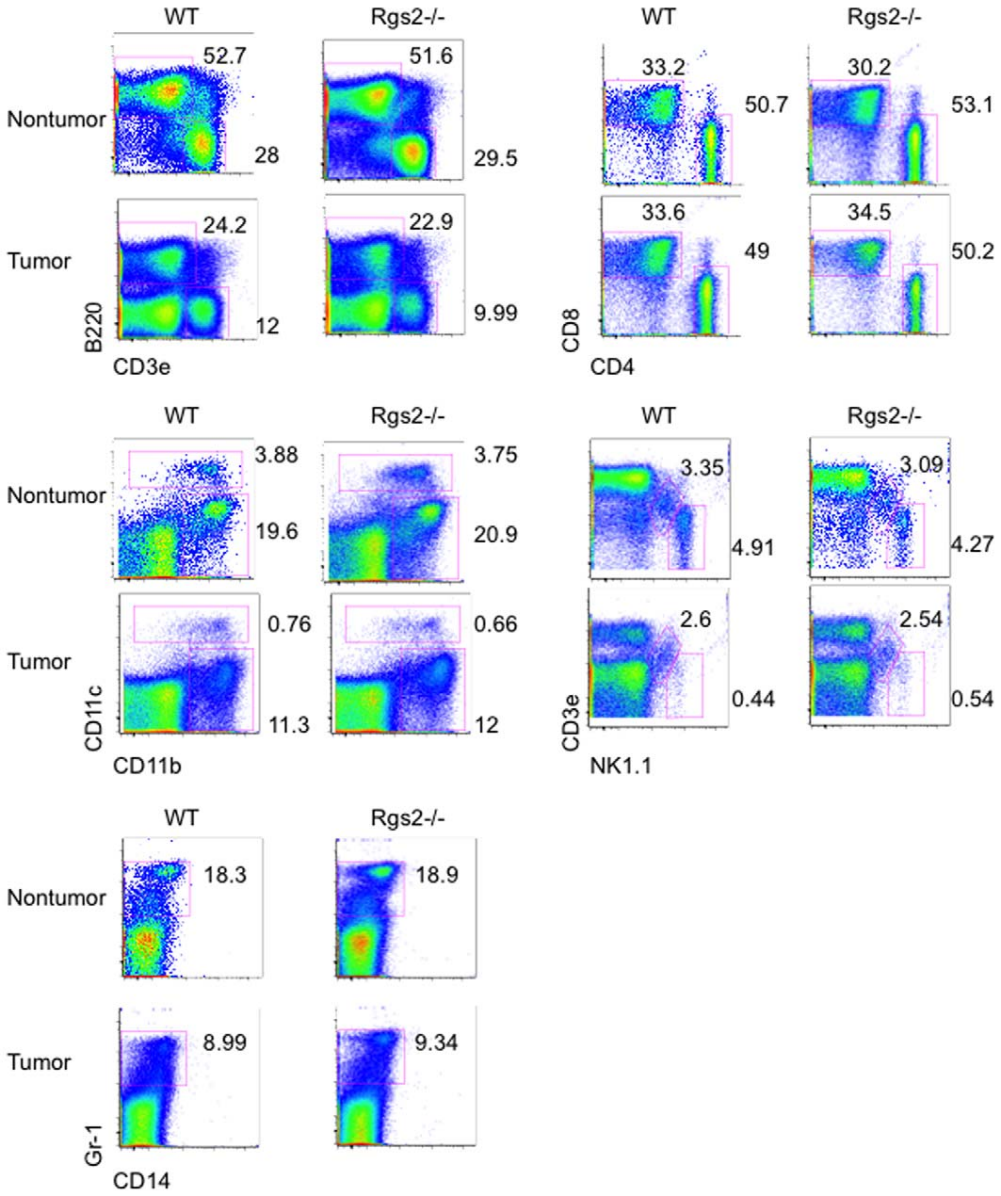
Lack of Rgs2 does not affect populations of mature leukocytes. Spleens were isolated from non-tumor bearing and 3LL tumor bearing WT and Rgs2−/− mice, processed into single cell suspensions, and labeled with the indicated antibodies, then analyzed by flow cytometry. Experiment was performed 3 times with 3–4 mice per group.

To examine the specific role of Rgs2 in MDSCs, we performed a reconstitution experiment. MDSCs were isolated from spleens of tumor bearing wild type or null mice by fluorescence activated cell sorting using antibodies against Gr-1 and CD11b. We achieved greater than 95% purity (data not shown). These MDSCs were co-injected with 3LL cells subcutaneously in the hindlimb of wild type mice, and tumor growth was measured over time (Figure 2B). We found that while wild type MDSCs were able to promote tumor growth, Rgs2−/− MDSCs lost their tumor promoting function, when compared to growth of the tumor cells alone (Figure 2B). Furthermore, we evaluated lung microvascular endothelial cells isolated from WT and Rgs2−/− mice, and found no difference in vascular tube formation, BrdU incorporation, or endothelial cell migration in response to serum stimulation between the two groups (Figure 3). In addition, Rgs2 deficiency did not affect the proportions of immature hematopoietic populations (data not shown) or mature hematopoietic populations (Figure 4). Together, these data support a specific role of Rgs2 signaling in MDSCs that is responsible for their tumor promoting activity.

### Tumors from Rgs2−/− mice exhibit decreased vascularization and increased cell death

**Figure 5:**
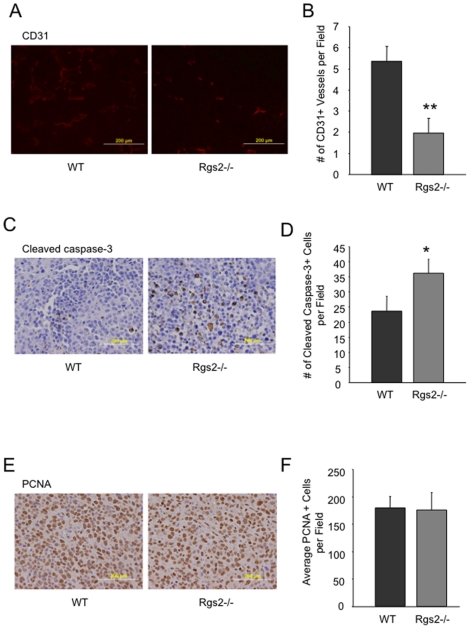
Tumors in Rgs2−/− mice exhibit decreased vascular density and increased cell death. (A), (C), (E) Sections from size matched 3LL tumors grown in wild type mice and Rgs2 null mice were stained for CD31, active caspase-3 and PCNA, respectively. Representative images are shown. (B), (D), (F) The numbers of CD31 positive vascular structures, active caspase-3 positive cells, and PCNA positive cells, respectively, were quantified in 10 randomly selected fields under microscopy. These experiments were repeated 3–4 times. ** p<0.005, * p<0.05.

MDSCs are known to infiltrate into tumors and promote tumor angiogenesis. Histological analysis of size-matched 3LL tumors from wild type and Rgs2−/− mice revealed a significantly lower vascular density, as measured by CD31-positive vessel structures, in tumors harvested from null mice than from wild type controls (Figure 5A–B). Similar results were observed when we used another vascular marker, von Willebrand factor (data not shown). Consistently, there is a significant increase in cell death, as indicated by cleaved caspase-3 staining in tumors from the null mice (Figure 5C–D). We did not see any significant difference in cell proliferation, as measured by PCNA staining, between the two groups (Figure 5E–F). These findings point to a pro-angiogenic function of Rgs2 in MDSCs in tumor growth and progression.

### Rgs2 plays no significant role in MDSC expansion and differentiation

**Figure 6:**
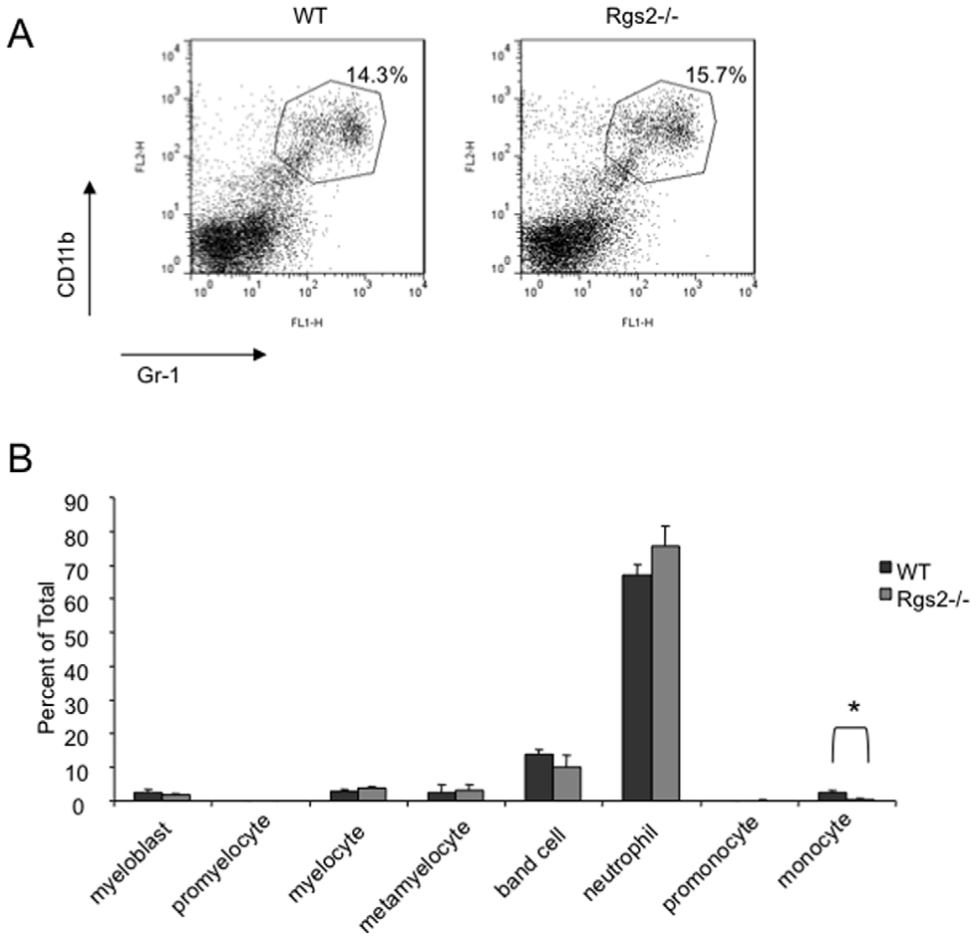
Rgs2 deficiency has minimal effects on MDSC expansion and differentiation. (A) Wild type and Rgs2−/− mice were injected with 1×10^5^ 3LL cells in the hindlimb, and 20 days later, spleens were isolated and analyzed by flow cytometry for Gr-1+CD11b+ MDSCs. This experiment was performed at least 3 times and the graphs shown are results from pooling of 3 mice per group. (B) MDSCs were isolated from spleens of tumor bearing Rgs2−/− and wild type mice using the MACS system, spun onto slides using a cytospin centrifuge, and stained. The slides were read by a hematopathologist in a blind fashion, and cells were categorized by morphology. This experiment was performed 4 times. * p<0.01.

**Figure 7:**
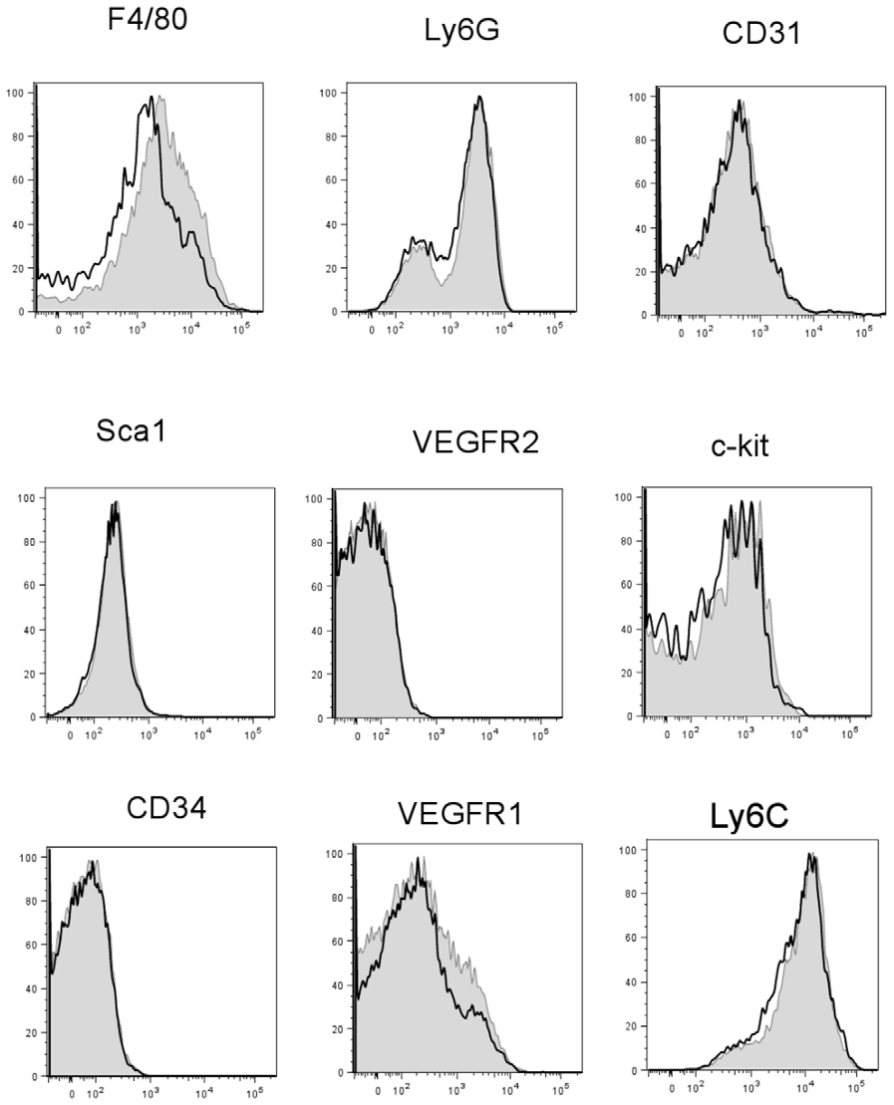
Analysis of cell surface molecules on Gr-1+CD11b+ MDSCs. Spleens were harvested from 3LL tumor bearing WT or Rgs2−/− mice between days 17–21 post-injection, processed into single cell suspensions, and labeled with antibodies against the indicated cell surface molecules. Representative plots are shown. Experiment was performed 3 times with 3–4 mice per group. Shaded = WT, open = Rgs2−/−.

MDSCs are elevated in tumor bearing hosts, and display different differentiation profiles compared to cells from non-tumor bearing hosts [4], [5], [6], [7]. Since Rgs2 is elevated in tumor derived MDSCs, we then determined whether Rgs2 has a role in MDSC expansion by analyzing spleens from 3LL tumor bearing wild type and null mice by flow cytometry. We found that spleens of both mice contained similar numbers of MDSCs (Figure 6A). Next, to determine if the lack of Rgs2 affects the proportions of the cells that comprise the heterogeneic Gr-1+CD11b+ fraction, we isolated Gr-1+CD11b+ cells from spleens of tumor bearing wild type and Rgs2−/− mice and scored them by morphology (Figure 6B). We observed a small decrease in the more monocytic MDSCs. However, no other cell types were different. Furthermore, we analyzed several cell surface molecules, such as Ly6C and Ly6G, on Gr-1+CD11b+ cells from wild type and Rgs2−/− mice by flow cytometry, and found no significant differences (Figure 7). These data indicate that it is less likely that Rgs2 plays a major role in expansion and differentiation of MDSC.

### Rgs2 upregulates MCP-1 in MDSCs

**Figure 8:**
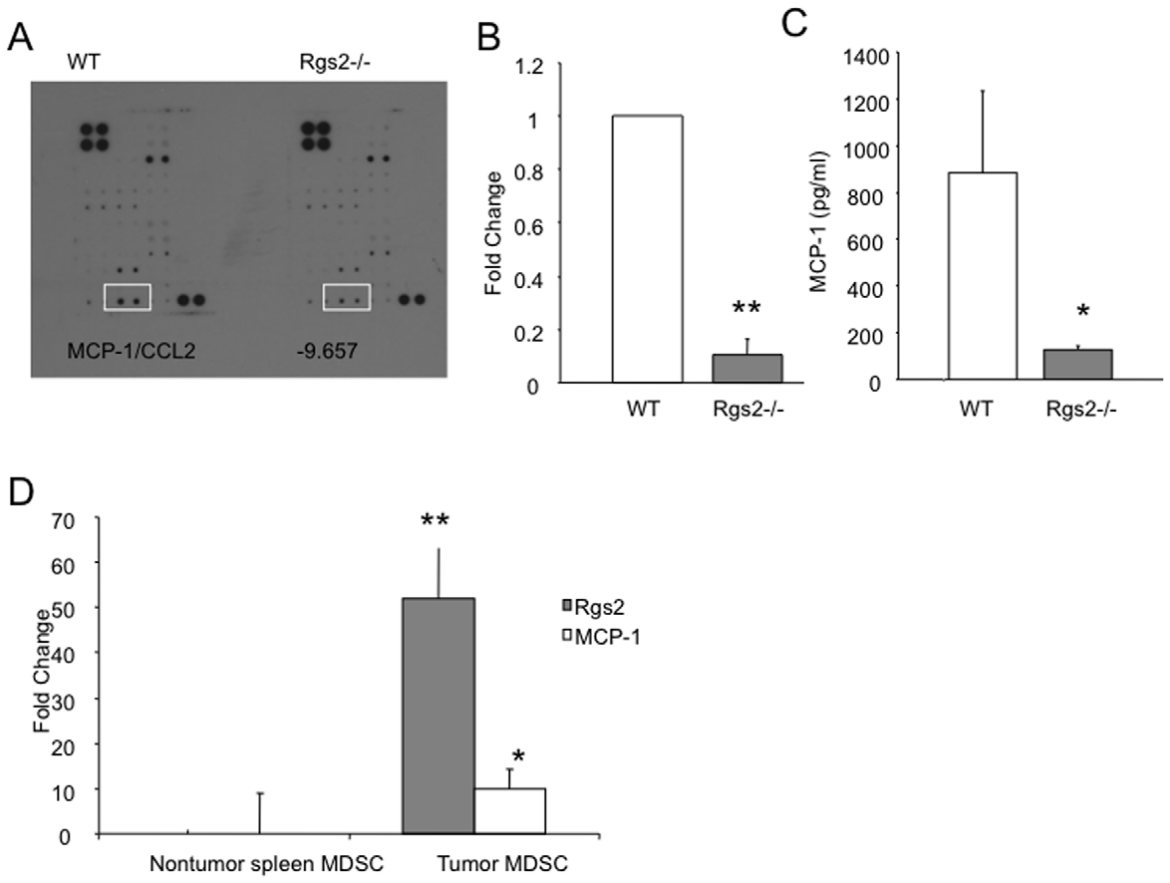
Rgs2 regulates MCP-1 expression in MDSCs. MDSCs were isolated from 3LL tumor tissues of wild type or Rgs2−/− mice by magnetic sorting after digestion of the tissues with hyaluronidase and collagenase. (A) Protein lysates from the isolated cells were analyzed using a cytokine array. Each cytokine is detected in duplicate, and intensity was determined using ImageJ software. Positive controls provided on the array were used for normalization. This experiment was performed twice. (B) RNA was extracted from the isolated MDSCs, and analyzed by real time PCR. Beta-actin was used as an internal control. This experiment was repeated 3 times. (C) MDSCs were isolated from tumors of wild type and Rgs2 null mice, and cultured for 48 hours. Culture medium was assayed for MCP-1 protein by ELISA. This experiment was performed in duplicate and repeated 3 times. * p<0.05, ** p<0.00005. (D) MDSCs were isolated from normal spleen and 3LL tumor tissues of wild type mice. Rgs2 and MCP-1 levels were measured by real time PCR. Beta-actin was used as an internal control. This experiment was repeated 2 times. * p<0.05, ** p<0.005.

A major function for MDSCs is secretion of growth factors, contributing to the cytokine milieu that promotes tumor growth. To determine if lack of Rgs2 affects cytokine production within these cells, we performed a protein cytokine array on the lysates of wild type and null MDSCs isolated directly from 3LL tumor tissue. We found a dramatic reduction of MCP-1 (∼9—10-fold) in Rgs2 null MDSCs compared to wild type MDSCs (Figure 8A). Using real time RT-PCR on RNA isolated from 3LL tumor tissue-derived MDSCs, we confirmed that, similar to the cytokine array, MCP-1 mRNA levels were significantly reduced in Rgs2 null MDSCs compared to wild type MDSCs (Figure 8B). Moreover, we measured MCP-1 protein levels by ELISA from media of cultured MDSCs purified from tumor tissues of wild type and Rgs2−/− mice for 48 hours. Consistently, we detected a dramatic reduction in the MCP-1 protein levels produced and secreted by Rgs2−/− MDSCs compared to wild type cells (Figure 8C).

In addition, we analyzed a potential correlation of Rgs2 and MCP-1 mRNA levels *in vivo* using freshly isolated MDSCs by real time RT-PCR. Consistent with an induction of Rgs2 in tumor derived MDSCs, there is a significant increase of MCP-1 levels in these cells compared to MDSCs isolated from non-tumor bearing mice (Figure 8D). These results support a positive role of Rgs2 in regulating MCP-1 expression in MDSCs. Together, the findings suggest that tumor conditions regulate Rgs2 expression, and Rgs2 regulates MCP-1 expression in MDSCs.

### Rgs2 mediates pro-angiogenic function in MDSCs through MCP-1

**Figure 9:**
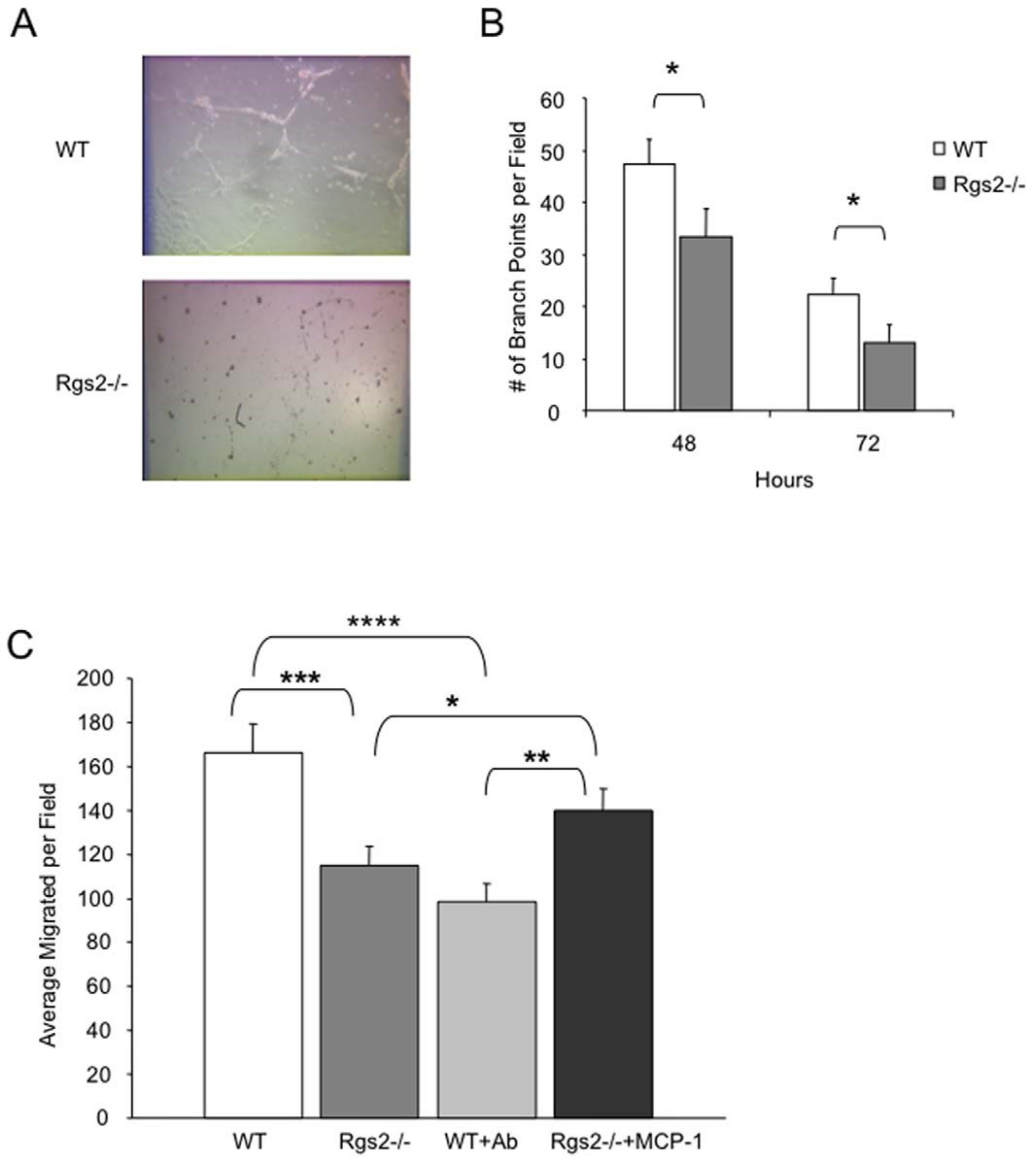
Angiogenic function of Rgs2 in MDSC is mediated through MCP-1. (A) and (B) Wild type and Rgs2−/− MDSCs were isolated from 3LL tumor tissues by magnetic sorting, and incubated overnight at 37°C. 80,000 HUVECs were plated in each well of a 48-well plate on top of Matrigel in the conditioned medium derived from the isolated MDSCs. Representative images are shown at 72 hours, and Vascular network branch points were scored at the times indicated. This experiment was performed in duplicate and repeated twice. (C) MDSCs were isolated from tumors of Rgs2−/− and wild type mice by magnetic sorting, and incubated overnight. Transwells containing 1×10^5^ HUVECs in the top chamber were added and allowed to migrate for 3.5 hours. MCP-1 neutralizing antibody (Ab) (1 ug/ml) was added to wild type cells or 1 ng/ml of recombinant MCP-1 was added to Rgs2−/− cells. This experiment was performed 3 times in duplicate. * p≤0.05, ** p<0.005, *** p<0.001, **** p<0.00005.

MCP-1 is a potent angiogenic factor, and we found that tumors in Rgs2−/− mice have decreased vascular density compared to tumors in wild type mice (Figure 5A–B). To determine whether decreased production of MCP-1 in the null MDSCs is responsible for defective angiogenesis associated with the Rgs2 null condition, we performed *in vitro* angiogenic assays. MDSCs isolated from tumor tissues of wild type or Rgs2−/− mice were cultivated overnight. Endothelial cells, (HUVECs) were then cultivated in the conditioned media from the MDSCs on Matrigel, which allowed vascular tube structures to form, and branch points were counted over time. There were significantly fewer vascular structures developed in the group treated with Rgs2−/− MDSC conditioned medium compared to those treated with wild type MDSC conditioned medium (Figure 9A and B).

Next, we assessed the ability of endothelial cells to migrate toward MDSCs. MDSCs were isolated from the tumor tissues of wild type and Rgs2−/− mice and cultivated overnight. Then, HUVECs were seeded in the top Transwell chamber and allowed to migrate toward the bottom chamber containing the MDSCs. In agreement with poor vascular tube formation induced by Rgs2 null MDSC conditioned medium, a significant reduction was observed in endothelial cell migration toward the null MDSCs compared to wild type cells (Figure 9C). Interestingly, addition of neutralizing MCP-1 specific antibody significantly inhibited wild type MDSC mediated endothelial cell migration. The number of migrated endothelial cells is not statistically different from the retarded migration observed toward Rgs2 null MDSCs (Figure 9C). Conversely, addition of recombinant MCP-1 with the Rgs2−/− MDSCs rescued defective angiogenic function (Figure 9C). Collectively, these data indicate that MCP-1 is responsible for Rgs2-mediated pro-angiogenic function in MDSCs.

## Discussion

MDSCs play a critical role in tumor progression through modulating the tumor microenvironment. They promote tumor growth at the primary site, as well as enhance metastasis [4], [6]. They have been linked to resistance to anti- angiogenic therapy in cancer [8]. Efforts focused at differentiating or attenuating the function of MDSCs have been promising, leading to improved immune response against the tumor and a better prognosis for the patient [2]. Clearly, identifying molecular mediators important for MDSC function will enhance our ability to better target these cells for cancer treatment. In this study, we identified a new mediator, Rgs2, that regulates pro-angiogenic function of MDSCs. We found that tumor conditions upregulate Rgs2 expression in MDSCs, and MDSCs lacking Rgs2 were no longer capable of promoting tumor growth in tumor reconstitution assays. Tumors in Rgs2−/− mice grew more slowly, and had less vascular density and increased cell death.

Furthermore, we identified the downstream molecular mediator responsible for Rgs2 mediated pro-angiogenic function in MDSCs. We found that Rgs2−/− MDSCs isolated from tumors produce drastically reduced levels of MCP-1 compared to wild type. While MCP-1 has been reported to promote migration of MDSCs [24], [25], production of MCP-1 by MDSCs has previously not been well studied. MCP-1 is a potent angiogenic factor. It promotes angiogenesis through indirect effects, such as through monocyte migration or induction of angiogenic factors such as VEGF and MMP9 [26], [27], [28], or by directly functioning on endothelial cells [22]. In this study, we show that wild type MDSCs secrete MCP-1, which promotes endothelial cell migration and vascular tube formation. Rgs2−/− MDSCs secrete much lower levels of MCP-1, which leads to reduced angiogenesis in tumors from Rgs2−/− mice.

Together, this study links the tumor promoting roles of Rgs2 in MDSCs to a secreted molecule, MCP-1. We show that hypoxia, commonly associated with solid tumors, upregulates Rgs2 expression in MDSCs, which leads to increased production of MCP-1. The MCP-1 then mediates the angiogenic effects. MCP-1 is produced mainly under pathological conditions, and is expressed by several cancer types [28], [29]. In a mouse model of breast cancer, treatment with neutralizing antibodies against MCP-1 enhanced survival and decreased metastasis [22], suggesting that targeting of this pathway in human cancer will likely prove beneficial. While it is uncertain how Rgs2 modulates MCP-1 levels, Rgs2 was predicted to encode a basic helix-loop-helix protein [30], [31]. Nuclear expression of Rgs2 was reported in several studies [16], [32], [33] including our unpublished data. These findings point to a function of Rgs2 in gene transcription. Future studies will be necessary to elucidate the mechanism involved.

In summary, our studies indicate that Rgs2 and MCP-1 are important molecules for the effector functions of MDSCs. Lack of Rgs2 in MDSCs abolishes their tumor promoting function, and leads to decreased levels of MCP-1, along with slower tumor progression and decreased vascular density in tumors. Thus, targeting Rgs2 and MCP-1 signaling may have important clinical implications, and lead to a better prognosis for cancer patients.
